# ACUTE CARE USE FOR OLDER ADULTS WITH DIABETES AND DEMENTIA IN A PRAGMATIC CLINICAL TRIAL OF PANEL MANAGEMENT

**DOI:** 10.1093/geroni/igad104.2960

**Published:** 2023-12-21

**Authors:** Brianna Morgan, Mauricio Arcila-Mesa, Crystalinda Rapozo, Oluwaseun Adeyemi, Joshua Chodosh

**Affiliations:** NYU Grossman School of Medicine, New York City, New York, United States; NYU Langone, New York City, New York, United States; NYU Grossman School of Medicine, New York City, New York, United States; New York University Grossman School of Medicine, New York City, New York, United States; NYU Grossman School of Medicine, New York City, New York, United States

## Abstract

Individuals with diabetes mellitus and Alzheimer’s disease and related disorders (DM-ADRD) face greater self-management challenges, over and under treatment, and more acute care episodes. Enhanced Quality in Primary Care for Elders with Diabetes-ADRD (EQUIPED-ADRD) is a pragmatic randomized controlled trial providing practice guidelines alone (control) compared to practice guidelines plus panel management (intervention) in primary care. Panel management includes patient self-management support and enhanced engagement and support for staff. Thirty-one primary care practices were randomized (14 control versus 17 intervention clinics). We assessed differences in emergency department (ED) use and hospitalizations in individuals with DM-ADRD comparing groups using univariate analysis. Included patients receiving care at randomized clinics were >65 years old, had DM, ADRD, an identified caregiver, and available utilization data. We tracked ED use and hospitalizations over 24 months using electronic health records. Panel management dose was number of phone calls. Among those having utilization data, 177 received panel management versus 349 who did not (total n=526). Patients were predominantly 75-84 years old (41.3%), female (59.9%), and non-Hispanic white (50.9%); 25.9% were Hispanic and 10.7% were non-Hispanic Black. 52.0% (n=92) panel managed patients had ED visits versus 41.8% (n=146) not panel managed [(p=0.027]; Odds Ratio (OR) 1.50; 95%CI: 1.05-2.16). Hospital use did not significantly differ between groups [35.0%; vs. 31.5%, respectively; p=0.418]. Increased panel management dose did not impact acute care use. These findings suggest panel management increases ED use. Whether this is due to greater detection of clinical problems or other issues needing attention requires further investigation.

